# Monitoring breast cancer progression through circulating methylated *GCM2* and *TMEM240* detection

**DOI:** 10.1186/s13148-025-01939-4

**Published:** 2025-07-21

**Authors:** Chin-Sheng Hung, Hsieh-Tsung Shen, Pei-Yu Wang, Chih-Ming Su, Wei-Wen Hsu, Kuan-Yu Chien, Cai-Sia Han, Li-Min Liao, Ruo-Kai Lin

**Affiliations:** 1 Department of Surgery, School of Medicine, College of Medicine, Taipei Medical University, Taipei, 11031 Taiwan; 2 Division of General Surgery, Department of Surgery, Taipei Medical University Hospital, Taipei, 11031 Taiwan; 3 Division of General Surgery, Department of Surgery, Shuang Ho Hospital, Taipei Medical University, New Taipei City, 23561 Taiwan; 4 The Ph.D. Program for Translational Medicine, Taipei Medical University and Academia Sinica, Taipei, 11031 Taiwan; 5 EG BioMed Co. Ltd., Taipei, 115011 Taiwan; 6 EG BioMed US Inc., Bothell, WA 98021 USA; 7 Division of Clinical Cariology and Endodontology, Department of Oral Rehabilitation, School of Dentistry, Health Sciences University of Hokkaido, Hokkaido, 061-0293 Japan; 8 Ji Yan Biomedical Co., Ltd., Taipei, 115603 Taiwan; 9 Department of Laboratory Medicine, Shuang Ho Hospital, Taipei Medical University, New Taipei City, 23561 Taiwan; 10 Department of Biostatistics, Health Informatics and Data Sciences, College of Medicine, University of Cincinnati, Cincinnati, OH 45267 USA; 11 Ph.D. Program in Drug Discovery and Development Industry, College of Pharmacy, Taipei Medical University, Taipei, 11031 Taiwan; 12 College of Medicine, Graduate Institute of Pharmacognosy, Taipei Medical University, Taipei, 11031 Taiwan; 13 Ph.D. Program in Clinical Drug Development of Herbal Medicine, Taipei Medical University, Taipei, 11031 Taiwan; 14 Master Program in Clinical Genomics and Proteomics, Taipei Medical University, Taipei, 11031 Taiwan; 15 Clinical Trial Center, Taipei Medical University Hospital, Taipei, 11031 Taiwan

**Keywords:** Breast cancer, *GCM2*, *TMEM240*, Circulating cell-free DNA, Methylation biomarker

## Abstract

**Background:**

Breast cancer is the most commonly diagnosed cancer and the second leading cause of cancer-related deaths in women worldwide. Approximately 20–30% of women diagnosed with early-stage breast cancer eventually develop metastatic disease. Current biomarkers, such as CA15-3 and CEA, detect metastasis in only 60–80% of cases, underscoring the need for improved diagnostic tools. This study investigates the potential of circulating methylated *GCM2* and *TMEM240* as biomarkers for noninvasive monitoring of breast cancer progression.

**Methods:**

In a prospective study conducted in Taiwan, 396 patients were enrolled, alongside a retrospective study of 134 plasma samples from Western populations. cfDNA was extracted, subjected to sodium bisulfite conversion, and the methylation levels of *GCM2* and *TMEM240* were measured using QMSP. Monte Carlo analysis assigned 70% of the dataset to a training set and 30% to a validation set, repeated 1000 times. Performance metrics such as sensitivity, specificity, and accuracy were averaged to ensure robustness, supporting the use of combined *GCM2* and *TMEM240* for monitoring treatment response and tumor burden.

**Results:**

The training set, consisting of 166 breast cancer patients (13.3% with recurrence or metastasis), was utilized to establish the biomarker detection cutoff. Validation in a separate cohort of 325 patients (20% with recurrence or metastasis) demonstrated superior performance compared to CA15-3 and CEA, achieving 95.1% accuracy, 89.4% sensitivity, 96.5% specificity, 86.8% positive predictive value (PPV), and 97.3% negative predictive value (NPV). Monte Carlo analysis of the training data revealed an average sensitivity of 95.7%, specificity of 90.3%, and accuracy of 91.5%, while validation data achieved 92.8% sensitivity, 89.5% specificity, and 90.3% accuracy across 1000 replicates. Positive cases were significantly associated with late-stage disease (*P* < 0.001), larger tumors (*P* = 0.002), distant metastasis (*P* < 0.001), and disease progression (*P* < 0.001). For monitoring treatment response and tumor burden, decreased methylation levels were observed in patients responding well to treatment, whereas increased levels were noted in cases of cancer progression or prior to metastasis.

**Conclusions:**

Overall, detecting methylated *GCM2* and *TMEM240* in plasma offers a novel, accurate, and noninvasive method for monitoring breast cancer progression.

**Supplementary Information:**

The online version contains supplementary material available at 10.1186/s13148-025-01939-4.

## Background

Breast cancer has now overtaken lung cancer as the world’s most commonly diagnosed cancer and is the second leading cause of cancer death among women worldwide [1–3] while also significantly contributing to global cancer mortality rates. International efforts are crucial for addressing the escalating burden of this disease, particularly in transitioning countries where the incidence of breast cancer is rapidly increasing and mortality rates remain high [4]. That said, breast cancer metastases, not the primary tumor, are responsible for more than 90% of cancer-related deaths [5]. Nearly 20–30% of patients with early-stage disease develop metastases over the disease course [6]. Advanced breast cancer, in which the cancer has metastasized to various organs, may not be completely curable with currently available treatments [7], primarily because of the development of multidrug resistance, which hampers treatment and prognosis [7].

Tumorigenesis involves the initiation and promotion of molecular abnormalities, including the activation of oncogenes and the inactivation of tumor suppressor genes (TSGs) [8]. The initiation and progression of cancer, which is conventionally considered a genetic disease, involve epigenetic abnormalities [9] such as DNA methylation defects and aberrant covalent histone modifications, which occur in all cancers throughout the natural history of tumor formation. These changes are detectable during early onset, progression, and ultimately recurrence and metastasis [9, 10].

There is an urgent clinical need for a relatively noninvasive system, such as blood testing, for monitoring treatment response and disease progression during cancer treatment. Cancer antigen 15-3 (CA15-3) levels are widely assessed for the early detection of recurrent breast cancer in current clinical practice. However, analyzing only CA15-3 as a tumor marker is insufficient for monitoring patients with breast cancer after surgical treatment [11]. Although clinically useful for some patients with metastatic breast cancer, the CA15-3 level has a sensitivity of only 60–70% [12–16]. Even the simultaneous use of serum markers CA15-3 and carcinoembryonic antigen (CEA) results in diagnosing metastasis in up to 60–80% of patients with breast cancer early [13–17], while according to our unpublished data, the simultaneous use of both CA15-3 and CEA resulted in the early diagnosis of metastasis in less than 50% of patients with breast cancer. No dynamic monitoring system is currently available for accurately measuring the complete treatment response and predicting recurrence in current clinical practice. Circulating cell-free DNA (cfDNA) in plasma can be used for the noninvasive sampling of cancer cells obtained from patients with breast cancer [18]. Cells (both cancerous cells and cells in the tumor microenvironment) release cfDNA through a combination of apoptosis, necrosis, and active secretion. Multiple genetic and epigenetic alterations are found in cfDNA [19]. For example, hypermethylated circulating glial cells missing transcription factor 2 (*GCM2*) has been reported as a noninvasive breast cancer-specific biomarker in both TCGA data and Taiwanese breast cancer patients, across all major breast cancer subtypes [20]. The *GCM2* gene encodes a transcription factor that is required for parathyroid development. Mutation of the C-terminal conserved inhibitory domain of *GCM2* can cause primary hyperparathyroidism [21]. Following surgery in breast cancer patients, there is a significant decrease in circulating methylated *GCM2*, indicating that circulating *GCM2* levels in breast cancer patients could serve as novel biomarkers for posttreatment monitoring, aiding in the detection of residual tumors [20]. Additionally, analysis of data from Taiwanese individuals in the TCGA database revealed that hypermethylation of transmembrane protein 240 (*TMEM240*) is associated with poor hormone therapy response in patients with breast cancer [22]. *TMEM240* encodes a transmembrane protein primarily found in the brain and cerebellum. In studies from various countries, including France, Germany, the Netherlands, Colombia, Japan, and China, mutations in *TMEM240* have been linked to spinocerebellar ataxia 21 (*SCA21*), leading to cognitive impairment and movement disorders, suggesting that the pathogenic mechanism underlying *SCA21* may involve early gliosis and lysosomal impairment caused by mutant *TMEM240* [23–27]. Hypermethylation of *TMEM240* has also been observed in colorectal cancer [28, 29]. Previously reported findings have indicated that circulating methylated *TMEM240* levels gradually decrease in Taiwanese patients with nonprogressive breast cancer and increase in those with disease progression, demonstrating a remarkable predictive accuracy of 96.1%. Notably, this predictive accuracy surpassed that of CA15-3 and CEA when assessing disease progression within a cohort of 57 patients [22]. Therefore, this study aimed to evaluate the potential of the simultaneous plasma detection of methylated *GCM2* and *TMEM240* in improving the prediction of residual disease and tumor progression in breast cancer patients. This evaluation was conducted with a large and highly diverse sample size, encompassing various racial groups.

## Methods

### Study design

A total of 38, 114, and 14 patients from TMU-H, TMU-SH, and Dx Biosamples, respectively, were selected to form the training group (earlier collection), while the validation group (latter collection) included 53, 152, 92, and 28 patients from TMU-H, TMU-SH, Precision for Medicine LLC, and Audubon Bioscience Co, respectively.

**Fig. 1:**
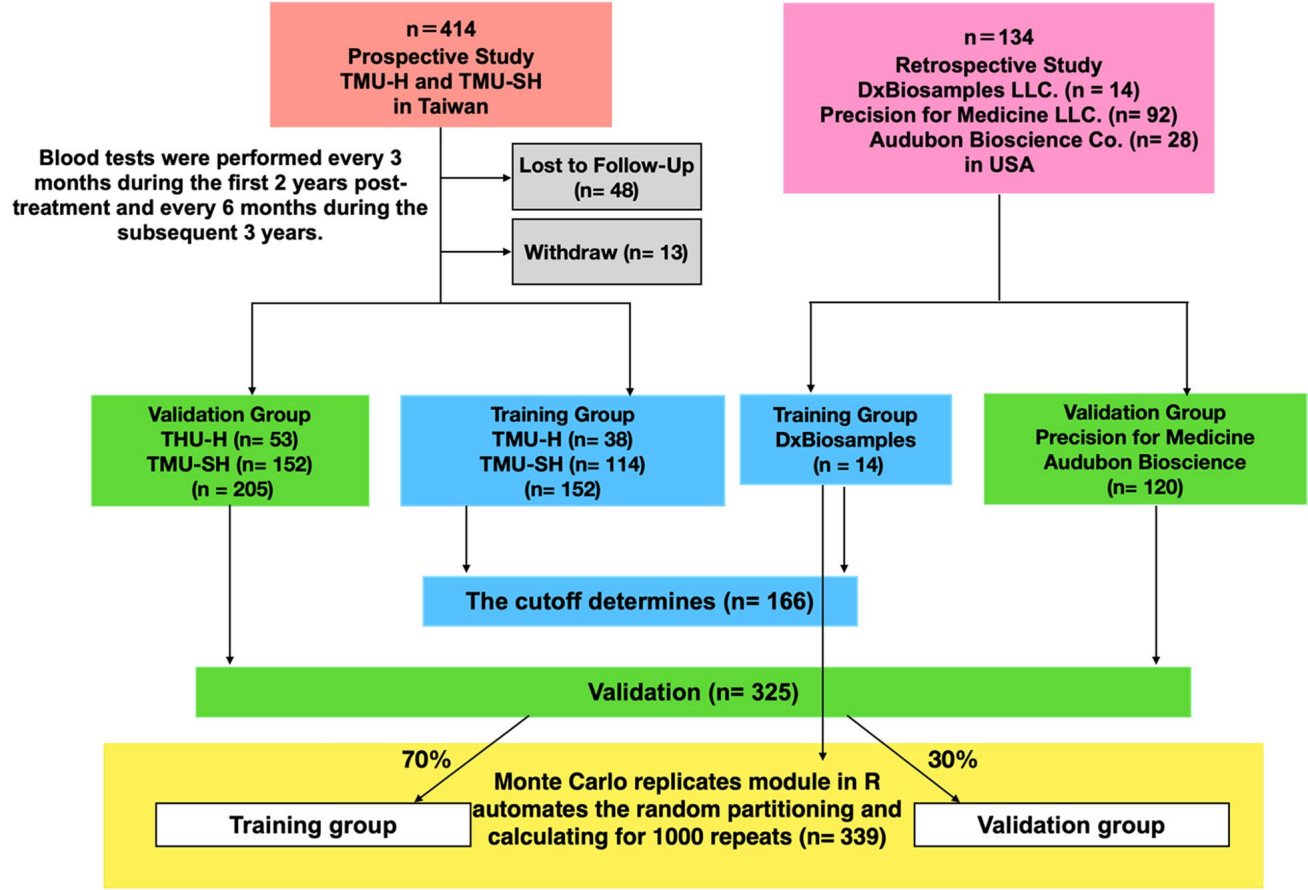
Study design and patient grouping in the establishment of the monitoring model

In addition to traditional cutoff determination and validation, this grouping facilitated the development of a monitoring model. We further used the Monte Carlo replicates module in R software to automatically partition the patient dataset into training (70%) and validation (30%) sets. The prospective and retrospective data were used to determine and validate the cutoff for the monitoring model. The designs of all the studies are detailed in Fig. 1.

### Patients and plasma collection

In a prospective study conducted in Taipei, Taiwan, we enrolled a total of 414 patients between 2016 and 2023. These patients were carefully monitored and included individuals with various stages of breast cancer, ranging from Stage 0 to Stage IV. The study participants were recruited from the Breast Medical Center at Taipei Medical University Hospital (TMU-H), as well as the Breast Medical Center and Division of Hematology/Oncology at Shuang Ho Hospital (TMU-SH). A total of 48 breast cancer patients were lost to follow-up after diagnosis. Additionally, 13 breast cancer patients withdrew from the clinical trial because of difficulties in or refusal of drawing an additional tube of blood.

Blood sample collection, cfDNA extraction, and cfDNA methylation analysis were conducted according to a predefined schedule, which included time points before surgery, 24 h after surgery, every 3 months during the initial 2 years posttreatment, and every 6 months during the subsequent 3 years. In addition to blood samples, clinical biomarkers and medical imaging data, including the levels of CA15-3 and CEA, and data from MRI, abdominal ultrasound, CT scans, bone scans, and breast ultrasound, were collected to monitor patients’ health and treatment progress comprehensively. The pathological diagnoses of these patients were confirmed through microscopic examination of patient specimens by two researchers. Sections of cancerous tissue and corresponding noncancerous tissues were assessed by a senior pathologist. Clinical data, including variables such as age, sex, tumor type, TNM tumor stage, menopausal state, and the statuses of tumor markers estrogen receptor (ER), progesterone receptor (PR), and human epidermal growth factor receptor 2 (HER2), were prospectively gathered from the medical records of TMU-H and TMU-SH. Prior to the collection of clinical data and samples, written informed consent was obtained from all patients. All clinical data, including the diagnosis of breast cancer, were obtained and confirmed by licensed medical doctors. The diagnostic assessments were further reviewed by Dr. Chin-Sheng Hung to ensure clinical accuracy.

We also conducted a retrospective study involving 134 plasma samples from Western populations. Among these, 120 samples were collected from US breast cancer patients, originating from two distinct sources: 92 from Precision for Medicine LLC. in Norton, MA, USA, and 28 from Audubon Bioscience Co. in New Orleans, LA, USA. An additional 14 plasma samples were obtained from Eastern European breast cancer patients through Dx Biosamples LLC in San Diego, CA, USA. The clinical characteristics of the enrolled breast cancer patients in the training and validation cohorts are shown in Supplementary **Tables S1** and **S2**.

### Circulating cell-free DNA extraction

Blood samples were collected via Streck BCT cfDNA tubes (Streck, La Vista, NE, USA), and a double centrifugation process was employed to isolate the plasma. cfDNA was extracted with the EG-Breast Blood Test-P1, EG cfDNA extraction Kit (EG BioMed Co. Ltd., Taipei, Taiwan) according to the manufacturer’s recommended protocol. The EG-Breast Blood Test-P1 is a qualitative qPCR-based assay designed to detect methylated cfDNA in human plasma using the EG BioMed testing system. It consists of three integrated components: (A) the EG cfDNA Extraction Kit, used to isolate cfDNA from plasma samples; (B) the EG Bisulfite Conversion Kit, used to perform bisulfite conversion and purification of the extracted cfDNA; and (C) the EG-breast blood test kit, used to detect and analyze methylated *GCM2* and *TMEM240* through methylation-specific PCR. First, one mL of plasma sample is incubated with proteinase K, SDS and magnetic beads for each reaction. After shaking, the tubes are centrifuged, and the magnetic beads are separated. The supernatant is carefully removed, and Extr-Wash is added to resuspend the magnetic beads. After another centrifugation and magnetic separation, ethanol is added, and the process is repeated. The samples are air-dried and transferred into nonmagnetic racks, and Extr-Elution is added to each microtube. After vortexing and spinning, the eluate containing the extracted cfDNA is transferred into fresh microtubes. After cfDNA extraction, the magnetic bead-containing microtubes are discarded, and the eluted cfDNA solution is cooled for subsequent processing. The entire volume is then used for the sodium bisulfite conversion reaction.

### Sodium bisulfite conversion of cfDNA

Sodium bisulfite conversion was conducted with the EG-Breast Blood Test-P1, EG bisulfite conversion kit (EG BioMed Co. Ltd., Taipei, Taiwan) according to the manufacturer’s recommended protocol. The sodium bisulfite conversion process involves incubating the mixture with a Bis-HQ solution. The Bis-HQ solution contains hydroquinone and sodium bisulfite and is used to protect DNA from degradation during the bisulfite conversion process. Next, magnetic beads and binding buffer are added, and the mixture is incubated. Then, microtubes containing the mixture are placed on a magnetic rack to separate the magnetic beads from the supernatant. Bis-Wash solution is added to each microtube, and the magnetic beads are resuspended on a shaker. After another round of magnetic separation, Bis-D solution is added to each microtube, and the magnetic beads are resuspended again. Following multiple wash steps and air-drying on a heater, the samples are transferred to nonmagnetic racks. Bis-Elution solution is added to each microtube, and the magnetic beads are resuspended. The eluate, containing bisulfite-converted DNA, is collected and transferred to fresh microtubes. The bisulfite-converted DNA mixture is then cooled before proceeding to the next step of qPCR analysis.

### TaqMan quantitative methylation-specific PCR

After DNA bisulfite conversion, the cfDNA methylation levels of *GCM2* and *TMEM240* were measured via TaqMan quantitative methylation-specific PCR (QMSP) with an EG-breast blood test kit and a Cobas LightCycler z480 (Roche, Germany) [20, 22]. The *beta-actin* (*ACTB*) gene was used as an internal control for input cfDNA. The QMSP conditions were as follows: preincubation at 95 °C for 10 min, followed by 50 cycles of amplification at 95 °C for 10 s and at 60 °C for 10 s. The entire bisulfite-converted cfDNA sample was equally divided into nine aliquots, with three replicates each for *GCM2*, *TMEM240*, and *ACTB* PCR assays. The specificity of the *GCM2* and *TMEM240* methylation end products was confirmed by bisulfite amplicon Sanger sequencing (Fig. S1). The primer and probe sequences, along with the reaction conditions used for the TaqMan qMSP assays, are listed in Table S3. These primers and probes were specifically designed to target regions that exhibit low methylation in normal tissue and high methylation in tumor tissue, as shown in our previous studies [22, 28, 30].

### Quality assessment of cfDNA methylation assays

The adequacy and quality of cfDNA input for each assay are evaluated using a methylation-specific PCR (MSP-PCR) assay targeting the internal control gene *ACTB*. The primers and probe for *ACTB* are designed to amplify both methylated and unmethylated cfDNA, serving as an endogenous control for assessing both cfDNA input quantity and extraction efficiency.

Based on prior clinical validation studies using plasma samples, a crossing point (Cp) value of *ACTB* below 35.5 is defined as the threshold for acceptable extraction efficiency. Cp values exceeding 35.5 indicate reduced cfDNA recovery, and values above 36.0 are generally associated with failed amplification of target methylation markers. Therefore, a Cp value of *ACTB* < 35.5 is required to ensure reliable assay performance.

Each assay run also includes an independent extraction positive control (ePC), which undergoes the complete workflow alongside the test samples. The *ACTB* Cp value of the ePC must likewise be below 35.5 to validate the extraction and assay quality for that batch.

### Statistical analysis

All the statistical analyses were performed with SPSS (SPSS Inc., Chicago, IL, USA). Pearson’s Chi-square test was used to compare circulating *GCM2* methylation level, circulating *TMEM240* methylation level, and clinical data, including age, sex, tumor type, TNM tumor stage, breast cancer subtype, and tumor status between groups. Comparisons of the hypermethylation and hypomethylation curves were performed with the log-rank test. *p* values of less than 0.05 were considered to indicate statistical significance. In addition to accuracy, other commonly used measures for evaluating classification, such as the receiver operating characteristic (ROC) curve and area under the curve (AUC), sensitivity, specificity, false positive rate, and false negative rate, are also reported.

### Creation of a monitoring model with the Monte Carlo replicates module

To further evaluate the overall performance of the circulating *GCM2* methylation level, circulating *TMEM240* methylation level, and internal control *ACTB* level in detecting disease progression, we used Su and Liu’s biomarkers [31]. This method can also accommodate an imbalanced distribution of outcomes resulting from the low prevalence of disease progression in the population. In our study, we randomly split the data into two datasets at a 7:3 ratio. Specifically, we used 70% of the original data to train a classification model and find the optimum threshold (i.e., that which led leading to the maximum AUC) for the classification. Then, with the optimum threshold and the estimated classification model, we used the remaining 30% of the data to examine how consistent the prediction and the true outcomes were for validation. We repeated this procedure 1,000 times and reported the average results. All the analyses were performed in R.

## Results

### Determining the cutoff value for detecting circulating methylated GCM2 and TMEM240 in plasma for predicting tumor progression

To assess the cutoff value for detecting circulating methylated *GCM2* and *TMEM240* in plasma for predicting tumor progression, we employed a training group comprising 14 Eastern European and 152 Taiwanese breast cancer patients. Of these 166 breast cancer patients, 13.3% experienced tumor recurrence or distant metastasis. Within this cohort, 56.0% of the participants were younger than 55 years, and all were female. Notably, 92.8% of the patients were diagnosed with the IDC subtype of breast cancer. A significant portion (77.1%) of the participants had stages II and III breast cancer, emphasizing the need for continuous monitoring to detect potential cancer progression events (Table S1).

Cutoff thresholds for *GCM2* and *TMEM240* were determined using Cp values in relation to clinical outcomes. A sample was classified as “POSITIVE” if it met any of the following criteria:

- *GCM2*: Cp value < 40.0 in at least one replicate and *TMEM240*: Cp value < 41.0 in at least one replicate; or
- *TMEM240*: Cp value < 41.0 in at least two replicates.

Samples not meeting these criteria were classified as “NEGATIVE.” These same thresholds were subsequently applied to the validation cohort to confirm their robustness and predictive utility.

**Fig. 2:**
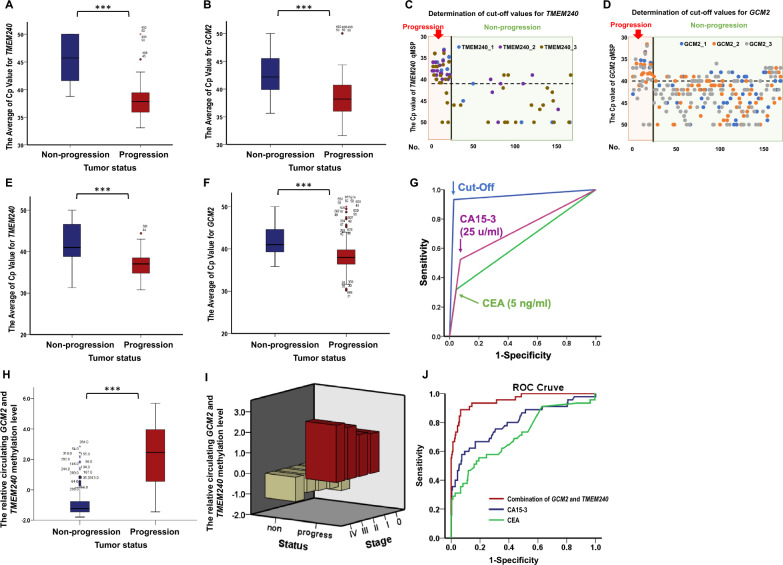
Distribution and performance of *TMEM240* and *GCM2* methylation levels in breast cancer progression. In the training group, box plots compare the average Cp values of *TMEM240* (**A**) and *GCM2* (**B**) between nonprogression and progression groups (166 subjects). Scatter plots display cfDNA methylation levels for *TMEM240* (**C**) and *GCM2* (**D**), showing progression patients in the orange area (left) and nonprogression patients in the green area (right). The Y-axis represents Cp values from qPCR, and the X-axis corresponds to individual patient numbers, with data based on three technical replicates per patient. In the validation group, bar charts show the average Cp values of *TMEM240* (**E**) and *GCM2* (**F**) for nonprogressive (left) and progressive (right) patients (325 subjects), with differences evaluated using the Mann–Whitney U test (***, p < 0.001). (**G**) ROC curves are based on classifications using the defined cutoff value for combined circulating *GCM2* and *TMEM240* methylation levels and are compared with CA15-3 and CEA levels for monitoring breast cancer progression. (**H**) Bar chart displaying combined *GCM2* and *TMEM240* methylation levels, calculated via the second Monte Carlo replicates module using Su and Liu’s method. (**I**) Methylated cfDNA of *GCM2* and TMEM240 in plasma across different stages at diagnosis. (**J**) ROC curves further compare the predictive performance of combined *GCM2* and *TMEM240* methylation levels against CA15-3 and CEA levels in monitoring breast cancer progression

By analyzing these samples, we found that the average Cp values in both the *TMEM240* and *GCM2* QMSP results were lower in the progression group than in the nonprogression group (Fig. 2A and B). A two-dimensional scatter plot was subsequently generated to visualize the data and determine the cutoff values. Subsequent analysis revealed that the progression group tended to have a *TMEM240* Cp value lower than 41.0 and a *GCM2* Cp value lower than 40.0 Conversely, in the nonprogression group, the Cp value of *TMEM240* tended to be greater than 41.0, whereas the Cp value of *GCM2* tended to be greater than 40.0 (Fig. 2C and D).

According to these cutoff values, the EG-breast blood test demonstrated an accuracy of 97.0%, a sensitivity of 81.8%, a specificity of 99.3%, a positive predictive value (PPV) of 94.7%, and a negative predictive value (NPV) of 97.3%.

### Clinical validation of circulating methylated GCM2 and TMEM240 levels for monitoring recurrence or progression in breast cancer patients

To further evaluate whether the combined detection of circulating methylated *GCM2* and *TMEM240* could be used to monitor for recurrence or progressive disease in breast cancer patients according to the defined cutoff values, we validated the system with the data from 120 breast cancer patients from the USA and 205 patients from Taiwan. Of the 325 patients, 66 (20.3%) experienced tumor recurrence or distant metastasis. Among the 259 patients without cancer progression, 55 (21.2%) showed no detectable methylated *GCM2* in their plasma across all three PCR replicates, and 200 (77.2%) had no detectable methylated *TMEM240*. In contrast, among the 66 patients with recurrence or metastasis, only 4 (6.0%) had no detectable methylation of either *GCM2* or *TMEM240* in all three replicates.

In this population, the proportions of individuals older than 55 years and those younger than 55 years were roughly equal. Similar to the training group, over 90% of the patients in the validation group were diagnosed with the IDC subtype of breast cancer. Additionally, 75.5% of these individuals were diagnosed with stage II or III breast cancer, indicating a clinical need for tracking potential progression in breast cancer patients (Table S2).

The raw data of the validation set were exported to an Excel file, and a box chart was created to visualize the average Cp values for *TMEM240* and *GCM2* (Fig. 2E and F). Overall, in the progression group, the Cp values of *TMEM240* and *GCM2* were lower than 40.0, whereas in the nonprogression group, the Cp value of *TMEM240* was greater than 40.0.

Analysis of the performance of these biomarkers revealed that the combined detection of circulating methylated GCM2 and TMEM240 via reagent analysis outperformed the levels of CA15-3 and CEA in terms of sensitivity, specificity, accuracy, PPV, NPV, and AUC; notably, the proposed system demonstrated a greater sensitivity for the progression group (Table 1). The combination of circulating methylated *GCM2* and *TMEM240* demonstrated an accuracy of 95.1%, a sensitivity of 89.4%, a specificity of 96.5%, a PPV of 86.8%, an NPV of 97.3%, and an AUC of 0.930. In comparison, use of the serum markers CA15-3 and CEA, commonly used to detect recurrence and metastasis in breast cancer patients in clinical practice, exhibited lower sensitivity (54.3 and 32.6%, respectively) and AUC values (0.737 and 0.639, respectively) (Fig. 2G).

**Table 1:** Performance of the combined detection of circulating methylated *GCM2* and *TMEM240* in the validation set

Test	N^a^	Sensitivity (%)	Specificity (%)	Accuracy (%)	PPV (%)	NPV (%)	AUC
*Overall*							
GCM2 & TMEM240	325	89.4	96.5	95.1	86.8	97.3	0.930
CA15-3	291	54.3	93.1	86.9	59.5	91.6	0.737
CEA	293	32.6	95.1	85.3	55.6	88.3	0.639
*Western Patients*							
GCM2 & TMEM240	95	92.3	89.3	90.5	85.7	94.3	0.904
CA15-3	74	55.2	75.6	67.6	59.3	72.3	0.654
CEA	75	34.5	89.1	68.0	66.7	68.3	0.617

^a^For some categories, the number of samples (n) was lower than the overall number analyzed because some clinical data of blood samples were unavailable

In addition, the performance of the combined detection of circulating methylated *GCM2* and *TMEM240* in a Western population was evaluated in this clinical study. Previous studies conducted in white populations have reported consistent findings, specifically that the combined detection of circulating methylated *GCM2* and *TMEM240* maintains a similar accuracy and sensitivity across different races. In this study, the combined detection of circulating methylated *GCM2* and the *TMEM240* demonstrated an accuracy of 90.5%, a sensitivity of 92.3%, a specificity of 89.3%, a PPV of 85.7%, an NPV of 94.3%, and an AUC of 0.904 for the Western population. In comparison, the use of markers CA15-3 and CEA yielded lower sensitivity (55.2 and 34.5%, respectively) and AUC values (0.654 and 0.617, respectively) (Table 1).

### Analysis of the monitoring model created by the Monte Carlo replicates module with Su and Liu’s classification method

To further validate the effectiveness of the combined detection of circulating methylated *GCM2* and *TMEM240* in plasma and create a real-time monitoring model, we employed Su and Liu’s classification method [31], an AUC-based classification approach suitable for multiple biomarkers. The average Cp value of *GCM2*, average Cp value of *TMEM240*, and average Cp value of *ACTB* were included in the model for calculation. This method can effectively handle imbalanced outcome distributions, which often occur due to the low prevalence of disease in the population. To ensure the robustness of our evaluation, we randomly divided the data into two sets at a 7:3 ratio. Specifically, 70% of the data were used to train the classification model and determine the optimal threshold (i.e., that which resulted in the maximum AUC) for classification. Using the optimal threshold and the estimated classification model, we subsequently examined the consistency between the predictions and true outcomes using the remaining 30% of the data. We repeated this procedure 1,000 times and reported the average results. All the processes for grouping and analysis were conducted in R.

The results demonstrated that Su and Liu’s method consistently exhibited high sensitivity, specificity, and overall accuracy across the 1,000 replicates. Specifically, the classification model generated using the training data exhibited an average sensitivity of 0.957, an average specificity of 0.903, and an average accuracy of 0.915. Furthermore, when the classification model was applied to the test data, the results showed an average sensitivity of 0.928, an average specificity of 0.895, and an average accuracy of 0.903 across the 1,000 replicates (Table 2). To investigate the correlation between elevated methylation levels of circulating *TMEM240* and *GCM2* and clinical parameter variations, patients were categorized into high (positive) and low (negative) methylation groups using cutoff values determined by the Monte Carlo replicates module and the method developed by Su and Liu. The positive patients were significantly more likely to be older (*P* = 0.002), have late-stage disease (*P* < 0.001), have larger tumors (*P* = 0.002), have distant metastasis (*P* < 0.001), and have disease progression (*P* < 0.001) (Table 3).

**Table 2:** Performance of Combined Methylation Markers Using Su and Liu’s Method

Parameter	Training Set (70%)	Validation Set (30%)
Nonprogressive (sample size, n)	182	77
Progressive (sample size, n)	52	23
Accuracy	0.915	0.903
Area Under the Curve (AUC)	0.964	0.912
Sensitivity	0.957	0.928
Specificity	0.903	0.895
False Positive Rate	0.097	0.105
False Negative Rate	0.043	0.072

**Table 3:** Association of clinical parameters with different levels of circulating methylated *GCM2* and *TMEM240* in patients

Characteristics	All N (%)^2^		Model by Su and Liu’s method^1^	
			Negative	Positive^3^
Overall	340	(100.0)	243(71.1)	96(28.3)
Age				
< 55 y/o	170	(51.1)	132(77.6)	38(22.4)
> 55 y/o	163	(48.9)	101(62.0)	62(38.0)**
Sex				
Female	330	(99.4)	233(70.6)	97(29.4)
Male	2	(0.6)	0(0.0)	2(100.0)*
Histological type	238			
IDC	220	(92.4)	180(81.8)	34(18.2)
ILC	9	(3.8)	6(66.7)	3(33.3)
Mucinous	5	(2.1)	5(100.0)	0(0.0)
Mixed	4	(1.7)	3(75.0)	1(25.0)
Stage at diagnosis	323			
0	1	(0.3)	1(100.0)	0(0.0)
I	48	(14.9)	42(87.5)	6(12.5)
II	190	(58.8)	142(74.7)	48(25.3)
III	56	(17.3)	40(67.9)	16(32.1)
IV	28	(8.7)	10(32.1)	18(67.9)***
Tumor size at diagnosis				
< 2 cm	97	(31.3)	78(80.4)	19(19.6)
> 2 cm and < 5 cm	178	(57.4)	130(73.0)	48(27.0)
> 5 cm	21	(6.8)	10(47.6)	11(52.4)**
On the chest wall/skin	14	(4.5)	8(57.1)	6(42.9)
Lymph nodes at diagnosis	319			
0	155	(48.6)	117(75.5)	38(24.5)
At least one	164	(51.4)	111(67.7)	53(32.3)
Metastasis at diagnosis	314			
No	278	(88.5)	216(77.7)	62(23.3)
Yes	36	(11.5)	9(25.0)	27(75.0)***
Subtype				
luminal A type	103	(44.0)	91(88.3)	12(11.7)
luminal B type	78	(33.3)	64(82.1)	14(17.9)
HER2 type	25	(10.7)	19(76.0)	6(24.0)
Basal-like (TNBC)	28	(12.0)	21(75.0)	7(25.0)
Tumor status	340			
Nonprogressive	259	(76.2)	227(87.6)	32(12.4)
Progressive	81	(23.8)	7(8.6)	74(91.4)***

^1^Data compared with the Pearson X2 test. *, *P* < 0.05; **, *P* < 0.01; ***, *P* < 0.001

^2^For some categories, the number of samples (n) was lower than the overall number analyzed because clinical data were unavailable

^3^Patients were classified as positive or negative based on a cutoff value determined using a logistic regression model developed according to the methodology of Su and Liu, with 5,000 bootstrap replicates

### Su and Liu-based logistic regression model for monitoring disease progression using methylated cfDNA in breast cancer

To evaluate relative methylation levels in individual patients, we developed a progression scoring model based on the methylation status of selected markers. Using a validation cohort, we applied a logistic regression model based on the methodology described by Su and Liu, incorporating 5,000 bootstrap replicates. Only models achieving 90% accuracy and false positive and false negative rates below 10% were retained, resulting in a total of 1,748 models. The average regression coefficients derived from these models are summarized in Table 4.

**Table 4:** Regression coefficients and cutoff value for cfDNA methylation-based progression score

	GCM2	TMEM240	IC_A	Cutoff Score
Bootstrap Coefficient	− 0.01973888	− 0.19878571	− 0.1467272	− 17.52127
Bootstrap Standard Error	0.0067762	0.0120404	0.045404	1.941738

The scoring formula is as follows:

Score = −0.01973888 GCM2—0.19878571 TMEM240—0.14672717 IC_A + 17.52127.

Decision Rule:

If Score > 0, the sample is classified as progression.

If Score 0, the sample is classified as no progression.

These "relative methylation levels" reflect the weighted contribution of each marker to the progression score. While the formula can be updated as more data become available, we expect future coefficient estimates to remain within the confidence intervals established by the bootstrap standard errors.

A box chart was created to visualize the relative methylation levels of circulating *TMEM240* and *GCM2*, which were calculated via the Monte Carlo replicates module and analyzed with Su and Liu’s classification method (Fig. 2H). To analyze the methylated cfDNA of *GCM2* and *TMEM240* in plasma across different stages at diagnosis, patients with disease progression exhibited high levels of methylated cfDNA of *GCM2* and *TMEM240* across all stages. In contrast, patients without disease progression showed low levels of methylated cfDNA of these biomarkers regardless of the stage (Fig. 2I). Overall, in the progression group, the relative methylation levels of *TMEM240* and *GCM2* were greater than those in the nonprogression group. The AUC of the model was 0.930. In comparison, the use of markers CA15-3 and CEA exhibited lower AUCs (0.737 and 0.639, respectively) (Fig. 2J).

### Monitoring the treatment response and tumor burden of breast cancer patients

**Fig. 3:**
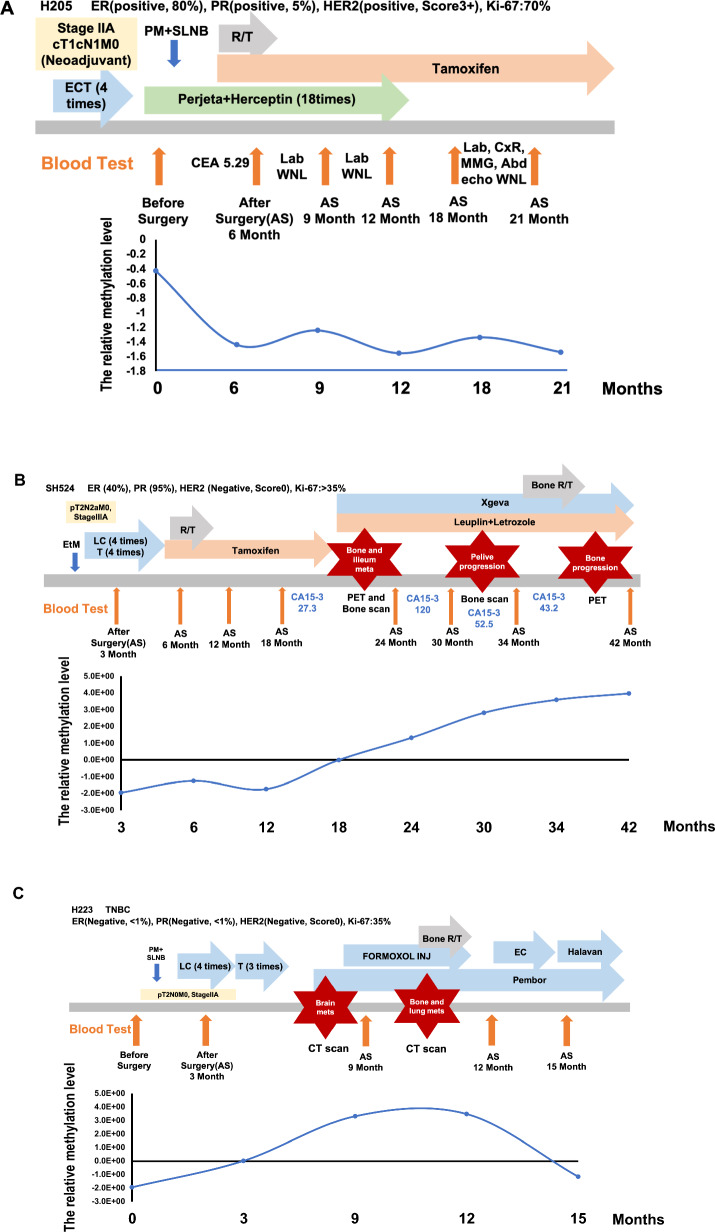
Real-time monitoring of circulating methylated *GCM2* and *TMEM240* in patients with breast cancer following treatment. Breast cancer patients with a good prognosis (**A**), with distant metastasis (**B**), and with distant metastasis who subsequently received adequate treatment (**C**). The Y-axis represents the relative methylation level, which was calculated with the Monte Carlo replicates module and analyzed with Su and Liu’s method. The X-axis represents months after treatment

We hypothesized that tumor burden in breast cancer patients could be dynamically assessed by monitoring circulating methylated *GCM2* and *TMEM240* following treatment. To evaluate this, we monitored their methylation levels every 3–6 months posttreatment, alongside routine blood tests for CA15-3 and CEA. Patient H205, who had a good outcome, showed a gradual decrease in the abnormal methylation of *GCM2* and *TMEM240* (Fig. 3A) in their plasma after treatment. In contrast, in the plasma of patient SH524, there was a clear and gradual elevation in the abnormal methylation levels of *GCM2* and *TMEM240* prior to the development of distant metastasis (Fig. 3B). The plasma of patient H223 demonstrated increases in the abnormal methylation levels of circulating *GCM2* and *TMEM240* as her breast cancer progressed and decreases when the patients receive adequate treatment (Fig. 3C).

## Discussion

In this study, we demonstrated that circulating methylated *GCM2* and *TMEM240* provide high diagnostic accuracy, sensitivity, and specificity for monitoring breast cancer progression. Compared to conventional serum markers CA15-3 and CEA, our assay significantly improves detection rates, particularly in early progression. This is consistent with prior literature emphasizing the limitations of protein-based biomarkers and the growing relevance of cfDNA methylation markers in cancer surveillance [12–17, 20, 32–34]. Prior studies have shown the potential of methylation-based assays for early detection and recurrence monitoring across various cancers, including breast, colorectal prostate, and lung cancer [35–37]. Our results extend these findings by demonstrating that the combination of *GCM2* and *TMEM240* methylation signatures achieves superior performance not only in Taiwanese patients but also in a Western cohort. This highlights the cross-ethnic applicability of our biomarkers, addressing a critical gap in cfDNA biomarker generalizability. Additionally, *GCM2* is frequently hypermethylated across both histological subtypes—such as invasive ductal carcinoma and invasive lobular carcinoma—and molecular subtypes, including luminal A, luminal B, HER2-enriched, and triple-negative breast cancer (Fig. S2). Early detection of an increase in tumor burden in patients with cancer recurrence and distant metastasis is crucial for timely treatment. As a key element of follow-up care and surveillance after the completion of primary breast cancer treatment, early detection aims to improve survival by identifying and treating recurrent disease while it is still potentially curable. This approach enables more effective salvage surgery and treatment, increasing the likelihood of a successful outcome [38], while also helping prevent the spread of cancer cells to multiple organs, keep the tumor in a manageable state, and potentially extend patient survival. When employed with a noninvasive approach, early detection not only reduces patient discomfort and inconvenience but also facilitates frequent testing. This increased comfort and convenience result in greater risk tolerance among patients, as they are more willing to accept potential risks associated with the test owing to the substantial benefits of early detection.

In the realm of breast cancer progression diagnostics, imaging techniques frequently reveal ambiguous lesions that may hinder accurate diagnosis. Blood tests can complement imaging techniques, enhancing diagnostic precision. However, the commonly used CA15-3 and CEA markers lack the necessary sensitivity [12–17], and there is a consensus against routine supplemental imaging for asymptomatic patients due to insurance coverage limitations. The emergence of circulating tumor DNA (ctDNA)-based minimal residual disease (MRD) tests holds great promise for enhancing sensitivity in detecting disease recurrence or progression. In our study, although dynamic changes in methylation appeared to parallel clinical progression, rather than consistently precede it, some early signals were noted. Because our cutoff values were established based on imaging-confirmed progression, biomarker positivity in our current model is aligned with radiologic findings. However, as shown in Fig. 3B and C, we observed that methylation levels of *GCM2* and *TMEM240* began to rise prior to radiographic evidence of progression. While the increase was modest, the upward trend—particularly the slope—suggests that methylation dynamics may serve as an early indicator of disease progression. Further prospective studies with predefined time points and longitudinal sampling will be essential to rigorously evaluate the lead time of these methylation markers in comparison to conventional clinical tools.

Surgical interventions for patients with breast cancer often result in postoperative artifacts, particularly with the increasing use of oncoplasty, which complicates imaging interpretation. This can lead to unnecessary biopsies, creating anxiety for patients. A highly sensitive blood test, such as the one we are developing, could mitigate these issues by providing a noninvasive monitoring tool, thus offering convenience and reassurance to postsurgical patients.

In clinical practice, for patients with stage II or III breast cancer, the detection of recurrence or metastasis through blood tests remains challenging, often requiring expensive procedures such as CT, MRI scans or bone scans to obtain a definitive diagnosis. In countries such as the USA, where such procedures are expensive, they are not routinely performed and are typically reserved for patients with overt symptoms. By this stage, however, it is likely that the disease has progressed to a point where therapeutic intervention may not be sufficiently effective. A sensitive blood test could address these limitations, providing an early and cost-effective means of monitoring for recurrence or metastasis, thus facilitating prompt and potentially life-saving treatments. Liquid biopsies are integral to this process, providing a noninvasive, real-time method that can be repeatedly used for thorough monitoring. Among various epigenetic markers, cfDNA methylation is the most extensively studied. The potential of the level of DNA methylation as a diagnostic indicator has been explored by examining the features of 5mC [39]. Sequencing-based, PCR-based, and microarray-based methods are commonly employed to detect these methylation changes in cfDNA. Recent research has revealed distinctive methylation patterns between cancer samples and noncancerous samples, reinforcing the role of DNA methylation as a valuable tool for detecting cancer [32–34].

However, DNA methylation assays have some limitations. When using EDTA blood collection tubes, it is essential to process samples within two hours to prevent leukocyte lysis and the release of genomic DNA (gDNA), which can compromise the accuracy of cfDNA-based analyses [40]. Circulating tumor DNA (ctDNA), a tumor-derived fraction of cfDNA, is typically present at low concentrations in plasma—often representing less than 1% of total cfDNA. In breast cancer, the proportion of methylated ctDNA is even lower, generally comprising less than 5% of total cfDNA [37]. The abundant background gDNA resulting from blood cell lysis caused by improper use of the assay has become a major obstacle to accurately measuring cfDNA [41]. While the use of cfDNA-specific collection tubes allows blood to be stored at room temperature for more than three days, these tubes are sixty times more expensive than standard EDTA tubes. Moreover, the capacities of blood collection tubes differ considerably in the preservation of blood samples. Therefore, suitable blood collection devices should be selected to minimize gDNA contamination and standardize blood sample processing to achieve more accurate and reliable clinical analysis of cfDNA [41], which contain blood stabilization reagents (preservation solutions) reported to inactivate virus activity [42], possibly significantly reducing biohazard risks compared with those of regular blood samples.

Additionally, cfDNA fragments are typically 166 bp long, matching the combined length of nucleosome-wrapped and linker DNA and indicating specific nuclease cleavage, and are released from cells through processes such as apoptosis. The quality of cfDNA can be affected by various preanalytical factors, such as the collection and storage method. Research shows that while these factors do not affect the size of cfDNA, they can influence its fragmentation patterns and end sequences, demonstrating the complexity of cfDNA analysis [43]. Therefore, the single-stranded, fragmented, and fragile nature of cfDNA, as well as the lack of protein wrapping and protection due to its origination from apoptotic or necrotic cells, complicate storage and increase the complexity of analysis.

Detecting cfDNA in plasma presents significant challenges because of its inherent characteristics. In healthy individuals, cfDNA is present in low quantities, approximately 0–10 ng/ml in plasma, whereas in breast cancer patients, the concentration increases to approximately 15 ng/ml [44]. Additionally, cfDNA has a short half-life, ranging from 16 min to 2.5 h, depending on various physiological and pathological factors [45]. These properties pose substantial difficulties in accurately detecting and quantifying cfDNA. The detection of tumor-derived cfDNA is technically challenging due to its low abundance in the bloodstream and its rapid degradation. This challenge is compounded by the release of cfDNA from noncancerous cells, which introduces substantial background noise and interferes with the identification of tumor-specific signals. These issues are particularly pronounced in early-stage cancer and in patients with minimal residual disease (MRD) following treatment, where tumor-derived cfDNA may be present at extremely low levels [46]. To overcome these limitations, highly sensitive and specialized analytical methods—such as digital PCR (dPCR), methylation-specific PCR (qMSP), and targeted next-generation sequencing (NGS) incorporating unique molecular identifiers (UMIs)—are required to enable accurate detection and quantification of tumor-associated cfDNA [30, 47, 48]. In addition, the use of IVD-grade reagent kits developed in a GMP-certified facility is strongly recommended prior to clinical application. These kits are produced under strict quality control to ensure lot-to-lot consistency, stability, and reproducibility, thereby supporting reliable and accurate cfDNA analysis across different runs and time points. Furthermore, standardized operating procedures implemented through automated liquid handling systems help reduce variability and improve assay robustness, addressing the inherent challenges of cfDNA handling and enabling more consistent clinical performance.

Additionally, hypermethylation of *TMEM240* has been shown to lead to the proliferation of breast cancer and colorectal cancer cells [22, 28]. Gene-specific demethylating approaches targeting *TMEM240*, such as CRISPR/dCas9-TET1-mediated DNA demethylation technology [49], may hold potential for treating patients with methylated circulating TMEM240 cfDNA. These methylation markers could also serve as companion diagnostics to guide therapeutic decisions.

The model developed in this study has potential for aiding in the development of personalized treatment plans by helping to identify patients who would benefit most from therapy and allow for better monitoring of treatment response. This targeted approach would enhance treatment efficacy and optimize breast cancer management.

## Conclusions

The model developed in this study demonstrated superior accuracy, sensitivity, specificity, PPV, and NPV compared to the clinically commonly used markers CA15-3 and CEA. Additionally, the methylation levels of *GCM2* and *TMEM240* can also reflect the treatment status of breast cancer patients in real time. Therefore, detecting these methylation biomarkers in plasma provides a novel, accurate, and noninvasive method for monitoring breast cancer progression.

## Supplementary Information

Additional file1

## Data Availability

No datasets were generated or analysed during the current study.

## Abbreviations

GCM2: Glial cells missing transcription factor 2
TMEM240: Transmembrane Protein 240
ACTB: Beta-actin
SCA21: Spinocerebellar ataxia 21
CA15-3: Cancer antigen 15-3
CEA: Carcinoembryonic antigen
cfDNA: Circulating cell-free DNA
QMSP: Quantitative methylation-specific real-time polymerase chain reaction
qPCR: Quantitative real-time reverse transcription polymerase chain reaction
PPV: Positive predictive value
NPV: Negative predictive value
TNBC: Triple-negative breast cancer
TCGA: The cancer genome atlas
