# Supplementary material for: Monitoring breast cancer progression through circulating methylated GCM2 and TMEM240 detection
Source: Clin Epigenetics. 2025 Jul 21;17:128. doi: 10.1186/s13148-025-01939-4 (PMC12281721; doi:10.1186/s13148-025-01939-4)
Supplement: Supplementary file 1 — Additional file1 [file 13148_2025_1939_MOESM1_ESM.docx]

**Monitoring Breast Cancer Progression Through Circulating Methylated *GCM2* and *TMEM240* Detection**

**Supplementary Tables and Figure
Table S1. The clinical parameters in recruited breast cancer patients of training group**

| Characteristics | Cases (N)^a^ | Percentage (%) |
| --- | --- | --- |
| Overall | 166 | (100.0) |
| Age |  |  |
| < 55 y/o | 93 | (56.0) |
| > 55 y/o | 73 | (44.0) |
| Sex |  |  |
| Female | 166 | (100.0) |
| Race |  |  |
| Asian | 152 | (91.6) |
| U.S. White | 14 | (8.4) |
| Histology type |  |  |
| IDC | 154 | (92.8) |
| ILC | 6 | (3.6) |
| Mucinous | 4 | (2.4) |
| Others | 2 | (1.2) |
| Original Stages |  |  |
| 0 | 1 | (0.6) |
| I | 21 | (12.7) |
| II | 111 | (66.9) |
| III | 17 | (10.2) |
| IV | 2 | (1.2) |
| Tumor status |  |  |
| Non-progression | 144 | (86.7) |
| Progression | 22 | (13.3) |
| ^a^For some categories, the number of samples (n) was lower than the overall number analyzed because clinical data were unavailable for those samples. | | |

**Table S2. The clinical parameters in recruited breast cancer patients of validation group**

| Characteristics | Cases (N)^a^ | Percentage (%) |
| --- | --- | --- |
| Overall | 325 | (100.0) |
| Country |  |  |
| U.S. | 120 | (36.9) |
| Taiwan | 205 | (63.1) |
| Age |  |  |
| < 55 y/o | 160 | (50.3) |
| > 55 y/o | 158 | (49.7) |
| Sex |  |  |
| Female | 315 | (99.4) |
| Male | 2 | (0.6) |
| Race |  |  |
| White | 95 | (29.9) |
| Asian | 212 | (66.7) |
| Black or African American | 10 | (3.1) |
| Multiracial | 1 | (0.3) |
| Histology type |  |  |
| IDC | 205 | (91.9) |
| ILC | 9 | (4.0) |
| Mucinous | 5 | (2.2) |
| Mixed | 4 | (1.8) |
| Original Stages |  |  |
| 0 | 2 | (0.6) |
| I | 47 | (14.6) |
| II | 188 | (58.4) |
| III | 55 | (17.1) |
| IV | 30 | (9.3) |
| Tumor size |  |  |
| 0 | 14 | (4.5) |
| < 2 cm | 91 | (29.0) |
| > 2 cm and < 5 cm | 175 | (55.7) |
| > 5 cm | 21 | (6.7) |
| Chest Wall/ Skin | 13 | (4.1) |
| Lymph Nodes Spread |  |  |
| No | 151 | (48.2) |
| Yes | 162 | (51.8) |
| Distance Metastasis |  |  |
| No | 277 | (90.2) |
| yes | 30 | (9.8) |
| Tumor status |  |  |
| Non-progression | 259 | (79.7) |
| Progression | 66 | (20.3) |
| ^a^For some categories, the number of samples (n) was lower than the overall number analyzed because clinical data were unavailable for those samples. | | |

**Table S3 List of primer sequences and conditions used in the present study**

| Gene | primer | 5’→3’sequences | Application | Size  (bp) | Tm ($℃$) |
| --- | --- | --- | --- | --- | --- |
| *BACTIN* | Forward  Reverse  Probe | TGGTGATGGAGGAGGTTTAGTAAGT  AACCAATAAAACCTACTCCTCCCTTAA  ACCACCACCCAACACACAATAACAAACACA | MSP-M | 132 | 60 |
| *GCM2* | Forward  Reverse  Probe | GAGATAGGGCGGAGTTTTTC  CTTAACCGCGATACTAAACGTT  TCCACCCGAACGACAACATCGACC | MSP-M | 105 | 60 |
| *TMEM240* | Forward  Reverse  Probe | TTTTTCGTTTATTATTACGATCGAC  CGACCCCGCCCGATATCCATAA  TTTAGAATTATGAAGATTATGGTGTTC | MSP-M | 81 | 60 |

**
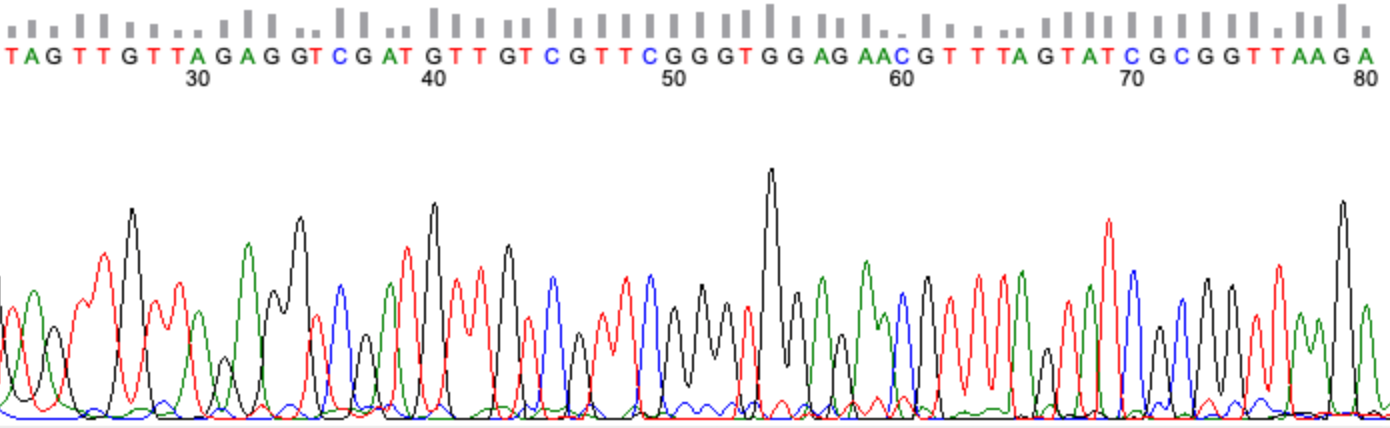
A**


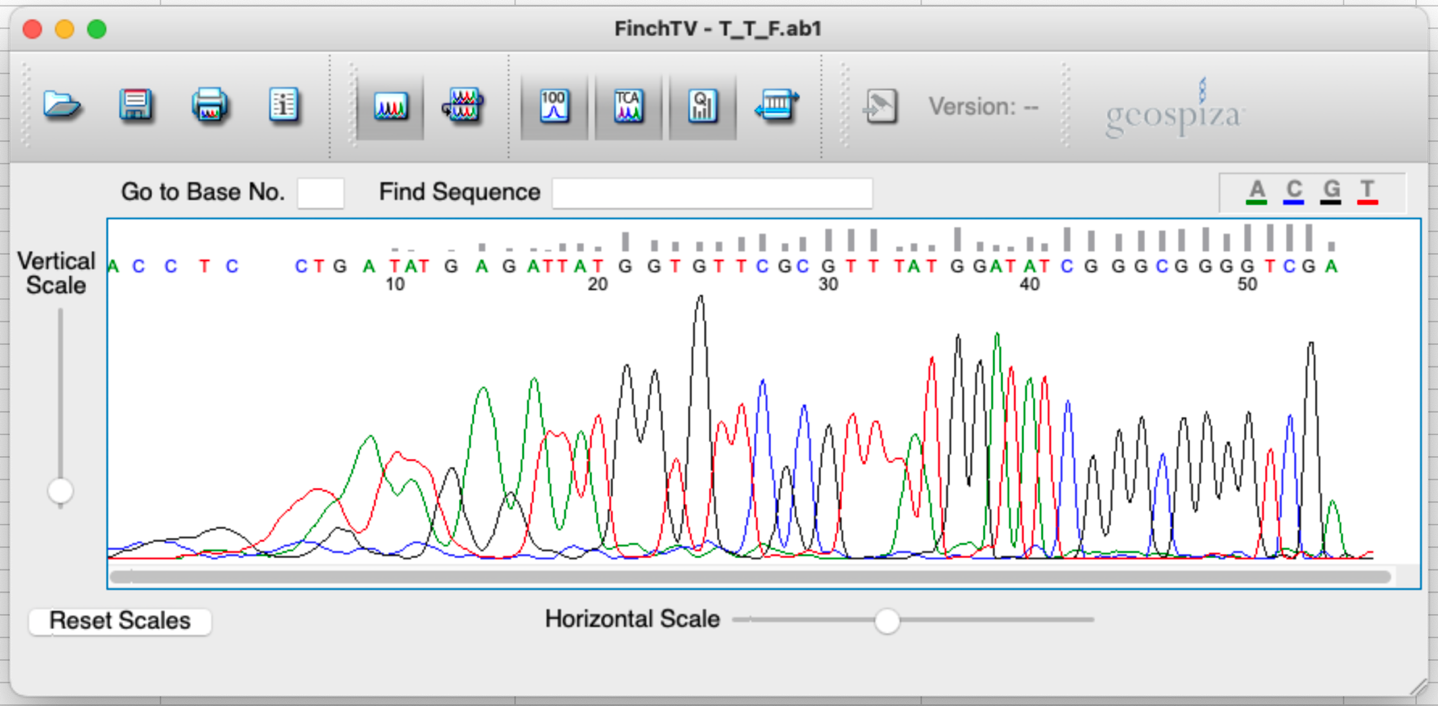
**B**

**Figure S1 Results of Sanger sequencing for *GCM2*(A) and *TMEM240*(B)**

**A

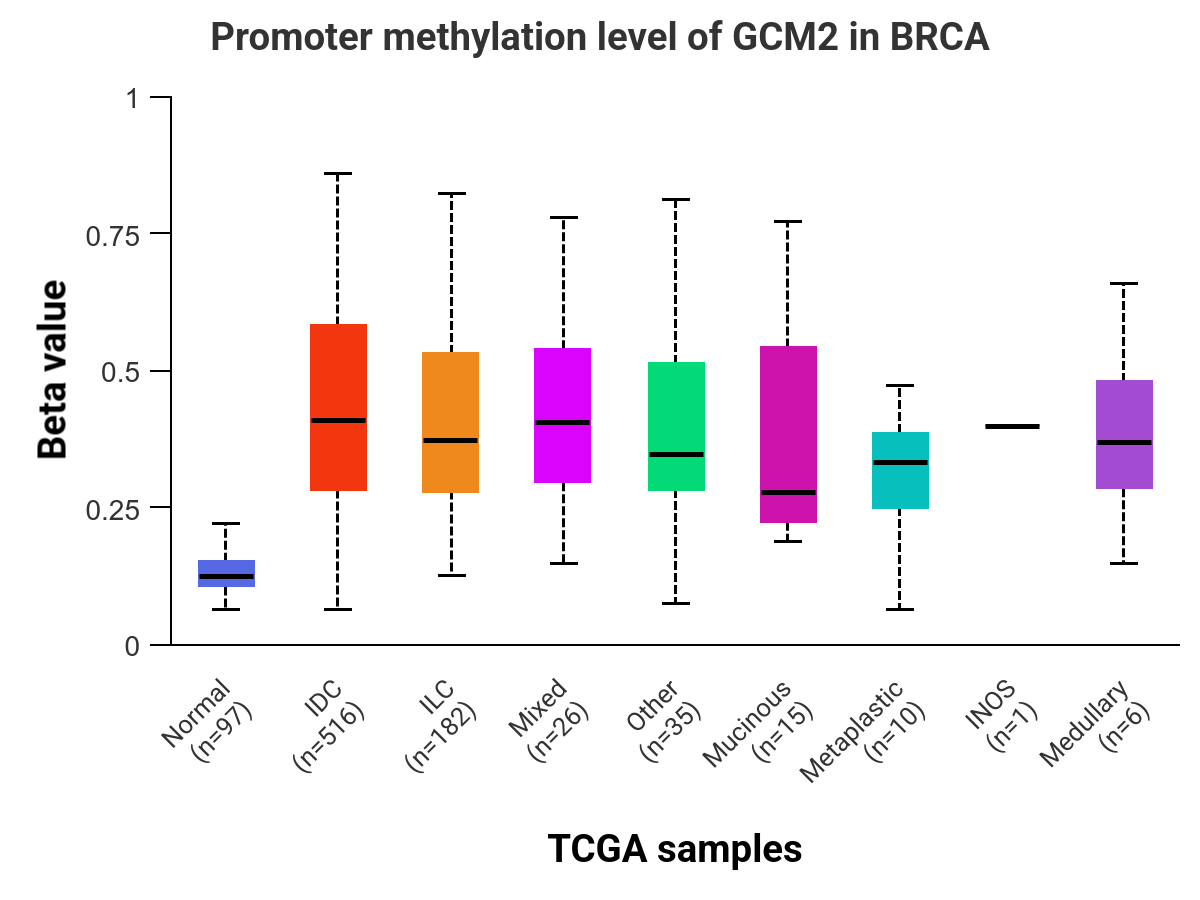
**

**B**

**
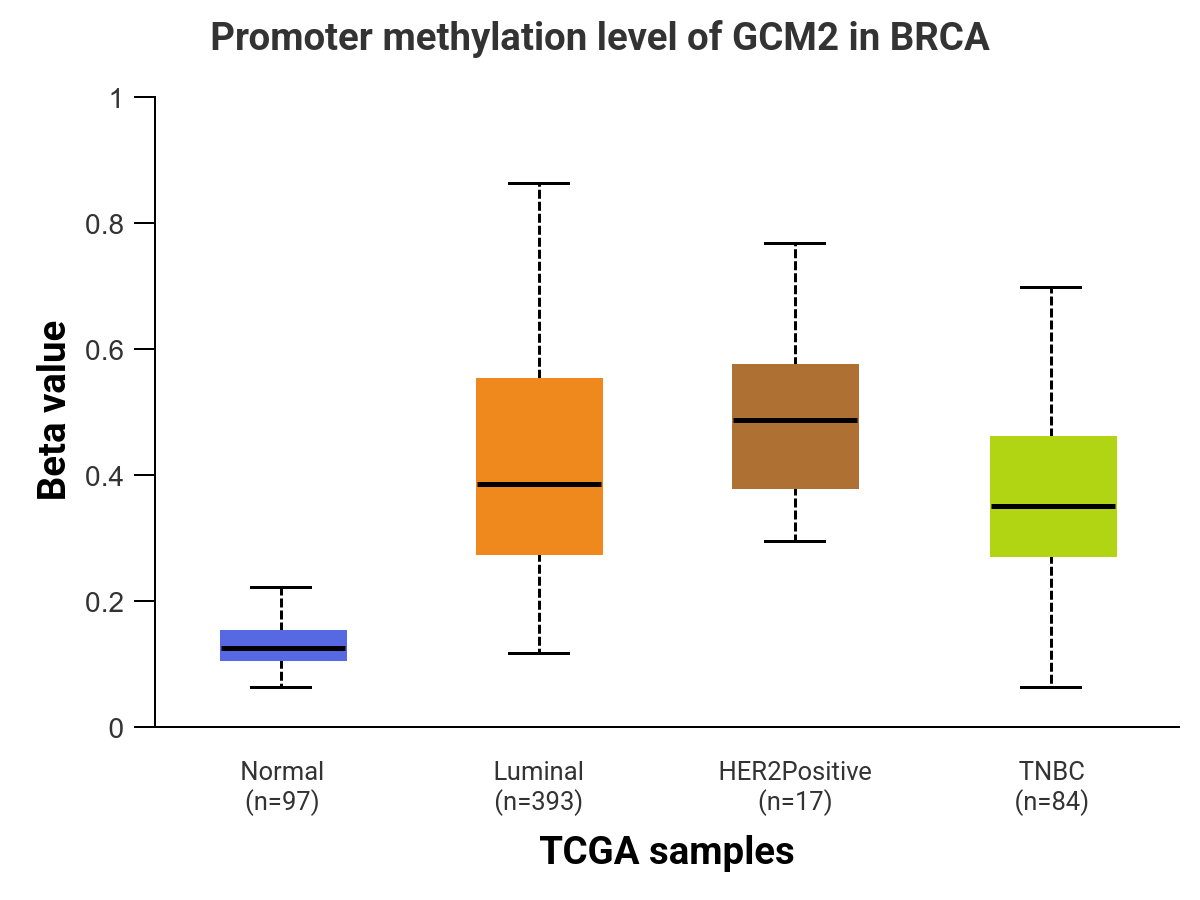
**

**Figure S2 Promoter Methylation Levels of GCM2 Across Breast Cancer Histological and Molecular Subtypes in TCGA Cohort.** (A) Histological subtypes; (B) Molecular subtypes.
